# Proliferation Rates of *Bovine* Primary Muscle Cells Relate to Liveweight and Carcase Weight in Cattle

**DOI:** 10.1371/journal.pone.0124468

**Published:** 2015-04-15

**Authors:** Chantal A. Coles, Jenny Wadeson, Carolina P. Leyton, Jason P. Siddell, Paul L. Greenwood, Jason D. White, Matthew B. McDonagh

**Affiliations:** 1 Department of Primary Industries Victoria, Discovery Technologies, Biosciences Research Division, Melbourne, Victoria 3083, Australia; 2 Department of Veterinary Science, University of Melbourne, Melbourne, Victoria 3010, Australia; 3 Cooperative Research Centre for Cattle and Beef Quality, University of New England, Armidale, NSW 2351, Australia; 4 New South Wales Department of Primary Industries, Beef Industry Centre of Excellence, Armidale, NSW 2351, Australia; Wageningen UR Livestock Research, NETHERLANDS

## Abstract

Muscling in cattle is largely influenced by genetic background, ultimately affecting beef yield and is of major interest to the beef industry. This investigation aimed to determine whether primary skeletal muscle cells isolated from different breeds of cattle with a varying genetic potential for muscling differ in their myogenic proliferative capacity. Primary skeletal muscle cells were isolated and cultured from the *Longissimus* muscle (LM) of 6 month old Angus, Hereford and Wagyu X Angus cattle. Cells were assessed for rate of proliferation and gene expression of *PAX7*, *MYOD*, *MYF5*, and *MYOG*. Proliferation rates were found to differ between breeds of cattle whereby myoblasts from Angus cattle were found to proliferate at a greater rate than those of Hereford and Wagyu X Angus during early stages of growth (5–20 hours in culture) *in vitro* (*P* < 0.05). The proliferation rates of myoblasts during early stages of culture *in vitro* were also found to be positively related to the liveweight and carcase weight of cattle (*P* < 0.05). Gene expression of *MYF5* was also found to be significantly down-regulated in WagyuX compared with Angus cattle (*P* < 0.05). These findings suggest that early events during myogenesis are important for determining liveweight and caracase weights in cattle.

## Introduction

Genetic and environmental factors that are involved in regulating muscle growth in cattle are of major interest to the beef industry. Increased muscling in cattle ultimately increases beef yield. Research is continuously being done to discover potential targets and mechanisms to increase beef yield. Myogenesis is first primarily regulated by four myogenic transcription factors from the myogenic regulatory factor (MRF) family, *Myf5*, *MyoD*, *MRF4* and *MYOG* [13]. During embryonic muscle development, muscle progenitor cells enter the myogenic lineage by first expressing *Myf5*, followed by *MyoD* [2, 8]. Myoblasts expressing *MyoD/Myf5* then differentiate and fuse with myoblasts to form multinucleated myotubes [14]. *MYOG* is expressed in myoblasts to promote differentiation [11]. *PAX7* is also a MRF expressed in satellite cells in postnatal skeletal muscle [14].

Satellite cells can be isolated from skeletal muscle and grown *in vitro*, whereby they undergo multiple rounds of cell division. A population of satellite cells from different genetic backgrounds in cattle may differ in their ability to be activated to enter the cell cycle and proliferate. This study isolated primary skeletal muscle cells from cattle, aged 6 months, that showed divergence in muscling EBVs; for the purposes of this study Angus were considered to have the highest muscling potential compared with Hereford and Wagyu X Angus (WagyuX) and determine differences in their ability to be activated and proliferate. We hypothesized that myoblasts derived from a high muscling breed of cattle will show the greatest rates of proliferation.

## Materials and Methods

Use of animals and the procedures performed in this study were approved by the New South Wales (NSW) Department of Primary Industries Orange Agricultural Institute Animal Ethics Committee (Approval Number ORA 08/005).

### Animals and Diets

Thirteen male calves (Angus (n = 4), Hereford (n = 5) and Wagyu X Angus (n = 4)) aged 6 months were grazed on New England perennial pasture (nitrogen fertilised Cocksfoot, Tall Fescue and Phalaris) until being transported to Northern Co-operative Meat Company (Casino, Australia) for slaughter.

### Sample Collection

Within 30 minutes of slaughter muscle samples (1–3 g) were collected. Samples were excised from the distal cranial region of the *Longissimus* muscle (LM) and incubated in 30 ml of chilled Hanks Buffered Saline Solution (HBSS) (Life Technologies, Carlsbad, CA). This muscle sample was placed in 30–40 ml of HBSS plus 10% (v/v) gentamicin (Life Technologies) cooled to 4°C.

### Muscle Digestion

Before muscle was digested, T75 (75 mm^2^) flasks were coated with 1% gelatin type B from bovine skin (Sigma-Aldrich, St. Louis, MO) as an attachment substratum. Muscle (1 g) was weighed and minced into fine pieces with a scalpel blade. The minced muscle was then added to a 50 ml tube containing enzyme mix (1.2 U/ml dispase (Life Technologies), 5 mg/ml collagenase (Life Technologies) and 5 nM CaCl_2_). The muscle sample was then left to incubate in 37°C, shaking at 150 x *g* for 30 minutes. Following this incubation tubes were centrifuged for 1 minute at 1,500 x *g* and the supernatant was added to growth media (Dulbecco’s Modified Eagle Medium (DMEM) plus 10% fetal bovine serum (FBS) (SAFC Biosciences, Melbourne, Australia), with 1% penicillin/streptomycin (Life Technologies) at 1:2 ratio). Another 2 ml of the enzyme mix was added to remaining muscle. Incubation at 37°C was repeated for another 30 minutes. This was repeated once more time with 2 ml of enzyme mix and then 1 ml, until no muscle was left in the tube. The muscle digest was filtered through a 100 μm^2^ cell strainer and centrifuged at 900 x *g* for 10 minutes at 4°C. The pellet was added DMEM plus recombinant fibroblastic growth factor (bFGF) (2.5ng/ml) (Biosource, San Diego, CA) in an uncoated petri dish and incubated for 60 minutes (to encourage fibroblast attachment). After this incubation, the media containing the myoblasts was transferred to a gelatin coated T75 flask and cultures were maintained in DMEM with 10% FBS, 1% penicillin/streptomycin and 5% (v/v) amphotericin B (Invitrogen) overnight in a humidified atmosphere of 95% air, 5% CO_2_ at 37°C.

### Immunocytochemical staining to determine myogenic cell populations

Cells at passage 2 were seeded 2 x 10^3^ cells per chamber of 8-well chamber slide (Nunc, Roskilde, Denmark) and cultured for 48 hours. Cells were then fixed in 4% formaldehyde containing 0.03% sucrose, washed in TBS. 0.05% Triton X-100:TBS was used to permeabilize cells before blocking overnight in 10% goat serum in TBS at 4°C. Cultures were washed in TBS:0.05% tween (TBS-T) and incubated in mouse monoclonal anti-desmin (Sigma-Aldrich, St. Louis, MO) in TBS overnight at 4°C. After washing with TBS-T, cultures were incubated with donkey anti-mouse Alexa488 secondary antibody (Life Technologies) for 90 minutes at room temperature in the dark. Cells were then washed in TBS-T, TBS and incubated in an aqueous mountant containing 4,6-diamidino-2-phenylindole (DAPI) (1μg/ml). Images were taken with an Olympus IX70 fluorescent microscope (Olympus, Australia) and Evolution VF monochrome digital camera (Media Cybernectics, Canada). Three fields of view were analysed for each animal to determine proportion of desmin positive cells to DAPI. A mean ± SEM was then calculated for each breed.

### Measurement of Cell Proliferation: xCELLigence System Technology

Using the xCELLigence system technology (Roche, Penzberg, Germany) and RTCA Software version 1.2 (Roche), cell index was used as a measure of cell proliferation of primary skeletal muscle cells isolated from each individual animal. Cell index is calculated from the relative changes in impendance at a certain frequency for a given time, allowing longitudinal assessment of culture growth over time. A 96-well xCELLigence E-plate (Roche) was plated out with 100 μl of DMEM with 10% FBS, 1% penicillin/streptomycin and 5% (v/v) amphotericin B and then placed in the xCELLigence system positioned in an incubator (humidified atmosphere of 95% air, 5% CO_2_ at 37°C) where a background read (1 sweep for 1 min) was performed using the RTCA software. Primary skeletal muscle cells from each animal at passage 2 (P2) were plated at 6.25 x 10^3^ cells/cm^2^ in triplicate into a 96-well xCELLigence E-plate so that the final volume per well was 200 μl. The E-plate was then left at room temperature for 30 min before being attached to the xCELLigence system. The schedule in the RTCA software was setup to measure impedance and thus cell index every hour for 120 sweeps. Cells reached confluence at 72 hours so data presented is calculated up to and inclusive of hour 72. Cell proliferation rate was calculated as the change in cell index over time (hours in culture).

### RNA extraction and Quantitative Real-time (qPCR)

Total cellular RNA was collected using the RNeasy Mini Kit (QIAGEN, Hamburg, Germany) for qPCR gene expression analysis of *MYF5*, *MYOD*, *MYOG* and *PAX7* using hypoxanthineguanine phosphoribosyltransferase (*HPRT)* as the housekeeping gene, refer Table 1 for primer sequences. First strand cDNA synthesis was performed on 1μg of total RNA using the Reverse Transcriptase – SuperScript III RT (Life Technologies). In a 96-well qPCR plate, 2 μl of cDNA (diluted 1:10) was added to 1 μl Forward Primer, 1 μl Reverse Primer, 10 μl H_2_O and 6μl Sybr Green Master Mix (total reaction volume = 20 μl) was added into each well. Each sample was run in triplicate for quantitative gene expression analysis. Plate was run on the Eppendorf thermal cycler (Eppendorf, New York, NY). Conditions for thermal cycling were as follows: 95°C for 10 min, 72°C for 1 min for 40 cycles and melting curve conditions. Relative gene expression of target genes was calculated against the reference gene (*HPRT*).

**Table 1:** Primer sequences used for qPCR analysis of myogenic gene expression in *bovine* primary muscle cells.

Gene	Primer SequenceForward 5’ → 3’	Primer SequenceReverse 5’ → 3’
MyoD	TTTGCCAGAGCAGGAGCCCCTC	TTCGAACACCTGAGCGAGCGC
Myf5	TGGCTGCTTTCGGGGCTCAC	GGTTGACCTTCTTCAGGCGTCTCC
Myogenin	CCGTGGGCGTGTAAGGTGTG	CCTCTGGTTGGGGTTGAGCAG
HPRT^1^	AGGACCCCTCGAAGTGTTG	TCCAGTTTCGCTAATGAC
Pax7	TGGTTCAGTAACCGCCGTGCC	TGCCCCCGTCTTGGGAGATAGTAG

^**1**^ Hypoxanthineguanine phosphoribosyltransferase.

The stability of the housekeeping gene in this study was determined as previously undertaken by co-authors [15]. Three housekeeping genes, hypoxanthineguanine phosphoribosyltransferase *(HPRT)*(Table 1), 18S ribosomal RNA *(18S)*(Forward 5’-3’ GTAACCCGTTGAACCCCATT, Reverse 5’-3’ CCATCCAATCGGTAGTAGCG) and glyceraldehyde 3-P dehydrogenase *(GAPDH)* Forward 5’-3’ACAGCGACACTCACTCTTCTACCT, Reverse 5’-3’ CCCTGTTGCTGTAGCCGAATTCAT) were measured against constant cDNA input (1μg of reverse-transcribed RNA from each well of 6-well plate (n = 3 for each animal) of confluent skeletal muscle primary cells. The effect of breed (Wagyu X Angus, Angus and Hereford) on reference gene expression was tested using General Analysis of Variance in Genstat 15.2 (VSN International Ltd, United Kingdom). Breed did not affect *HPRT*, *18S* or *GAPDH* gene expression (*P >* 0.05). The variation (standard error of mean (SEM)) in the gene expression of housekeeping genes for each breed was found to be lowest for *HPRT* (SEM ≤ 0.230) followed by *GAPDH* (SEM ≤ 0.413) and *18S* (SEM *≤* 0.939), therefore *HPRT* was considered to be the most stable housekeeping gene.

### Statistical Analyses

Statistical analyses used the ANOVA function of Genstat 15.2 (VSN International Ltd, United Kingdom). Breed was fitted as a fixed effect. Results are presented as means ± standard errors of the mean. A value of *P <* 0.05 was considered to be statistically significant.

## Results

### Determination of myogenic cell population

**Fig 1:**
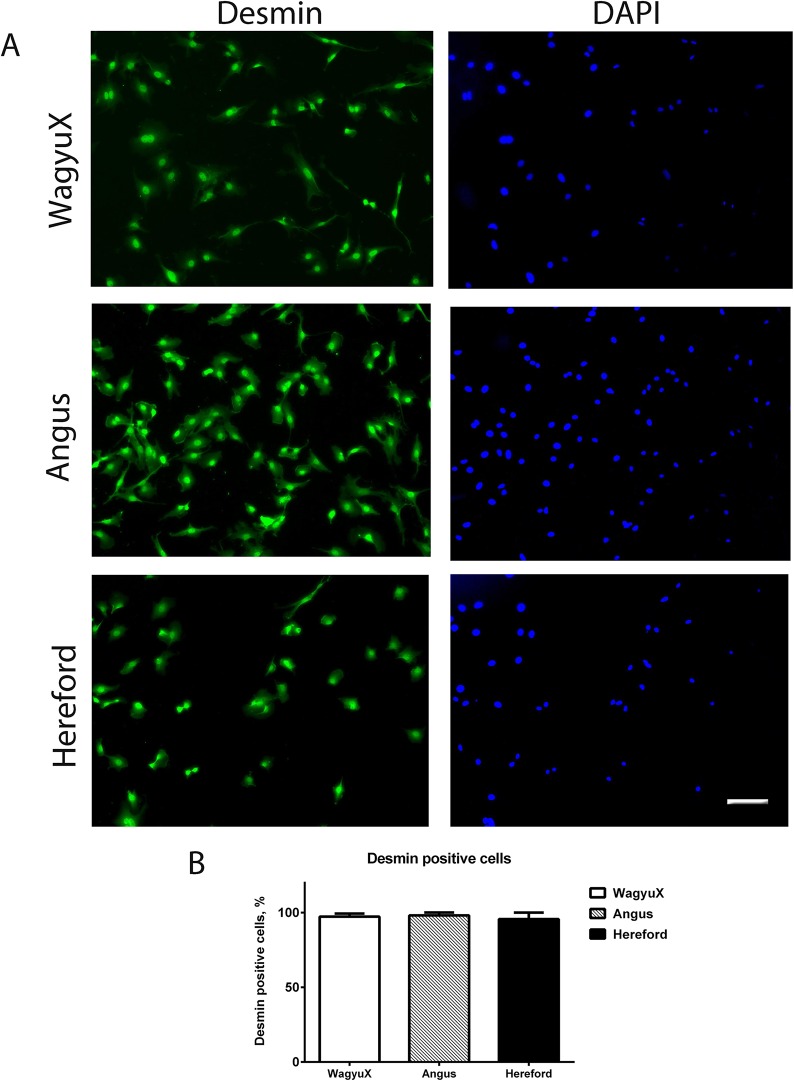
Determination of myogenic cell population. A. Expression of the myogenic marker desmin and nuclei (DAPI) in undifferentiated primary skeletal muscle cells from WagyuX, Angus and Hereford cattle. B. Mean ± SEM proportion of the total number of cells positive for desmin (myogenic cells). A good proportion of cells were positive for desmin for all breeds. There was no difference in proportion of desmin positive cells between WagyuX (97.33 ± 2.0), Angus (98.12 ± 2.0) and Hereford (95.67 ± 4.3). Scale bar is equal to 60μm.

Primary skeletal muscle cells were stained for the myogenic marker desmin and nuclei was stained for DAPI to determine proportion of desmin positive (myogenic) cells (Fig 1A). There was no difference in the proportion of desmin positive cells between WagyuX (97.33 ± 2.0), Angus (98.12 ± 2.0) and Hereford (95.67 ± 4.3) (Fig 1B). The proportion of desmin positive cells was greater than 95% for each breed.

### Proliferation of *bovine* Primary Skeletal Muscle Cells Under Myogenic Conditions Differs Between Breeds of Cattle

**Fig 2:**
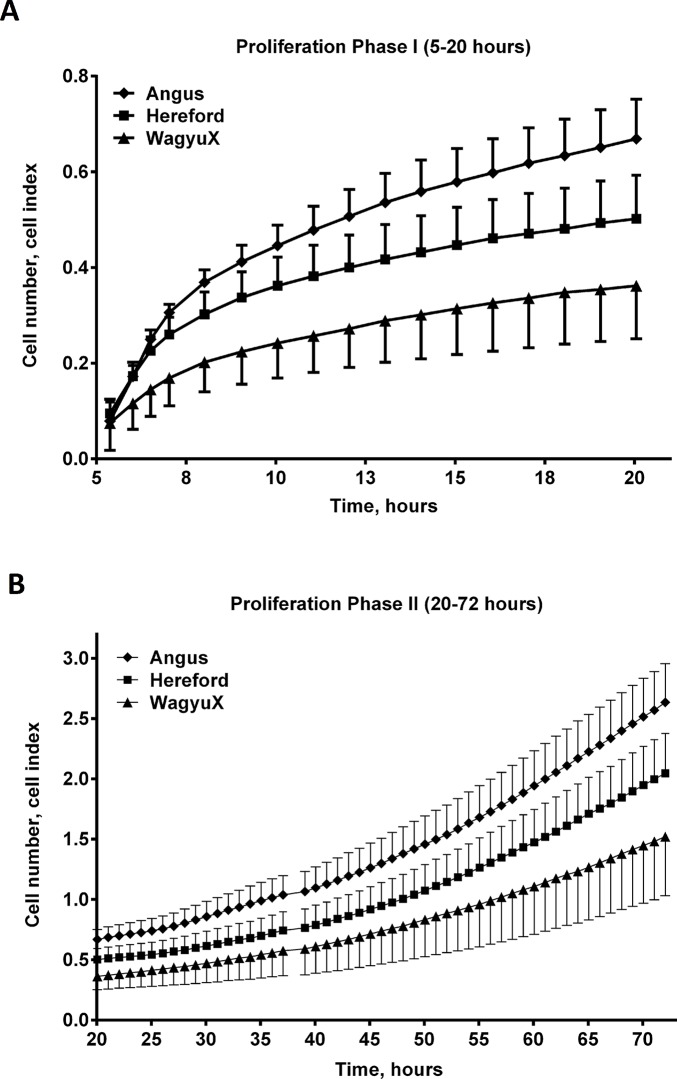
Angus primary muscle cells grow faster than primary skeletal muscle cells derived from Hereford and WagyuX cattle. The number of cells (cell index) was measured in culture using xCELLigence technology during two defined phases of growth. A. Proliferation Phase I (5–20 hours in culture) and B. Proliferation Phase II (20–72 hours in culture). (NOTE: Standard error bars in A and B are displayed as either positive or negative SE values only so the means are clearly visible and there is no overlap in error bars).

Proliferation of primary skeletal muscle cells was higher in Angus cattle, compared with Hereford and WagyuX cattle. Fig 2 demonstrates the average proliferation (cell index over time (hours in culture)) for each breed from xCELLigence plate readings. Cell index is a measure of cell density within each well and cell proliferation rate is the slope of the curve of best fit from cell index recordings within a given time frame (for example 5–20 hours).

**Fig 3:**
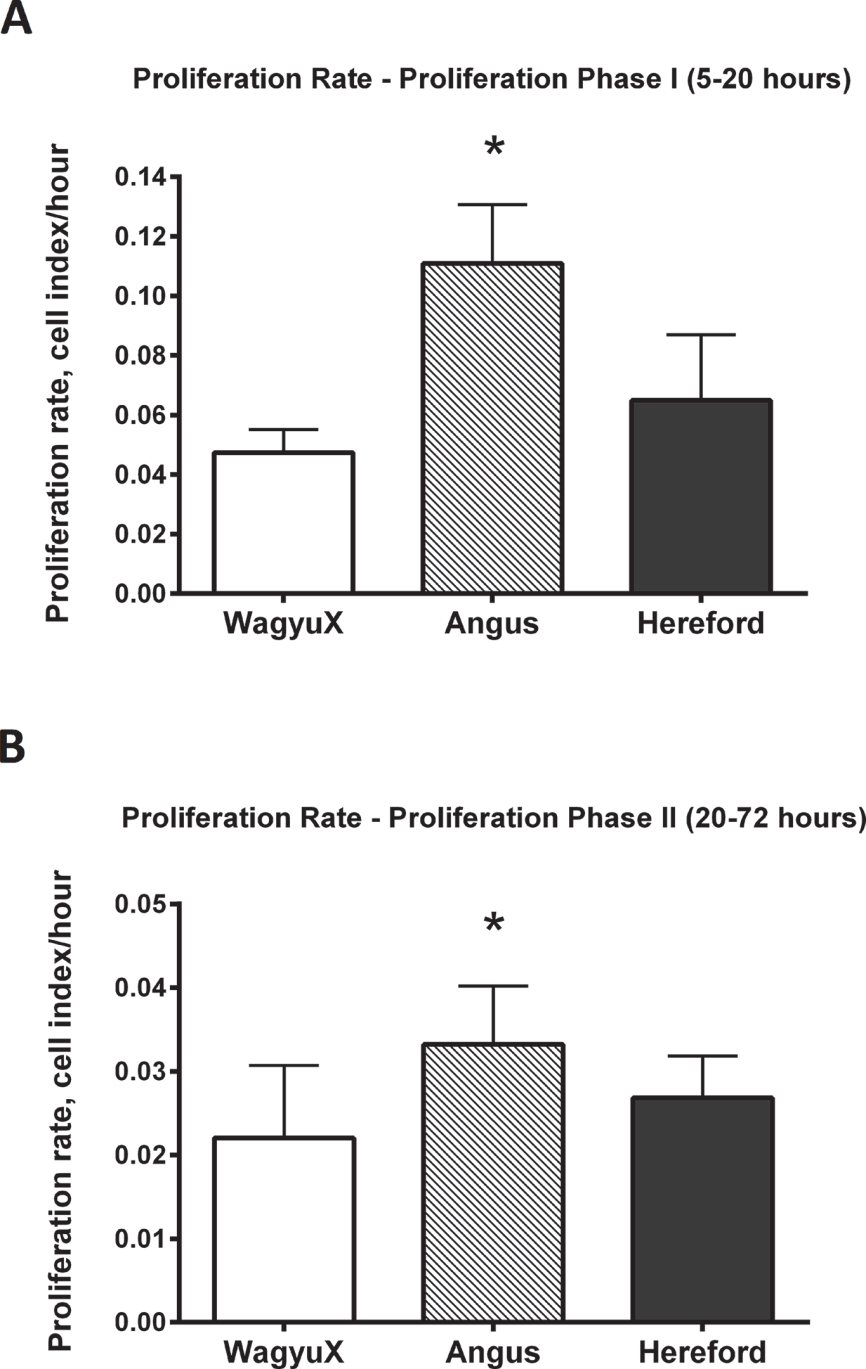
Angus primary skeletal muscle cells proliferate faster than cells from Hereford and WagyuX skeletal muscle. The mean rate of proliferation (cell index/hour) for each culture from WagyuX (n = 4), Angus (n = 4) and Hereford (n = 5) ± SEM for A. Proliferation Phase I (5–20 hours in culture) and B. Proliferation Phase II (20–72 hours in culture). Error bars represent SEM.

Proliferation rates of primary muscle cells were classified into two major growth phases, *Proliferation Phase I* and *Proliferation Phase II*. *Proliferation Phase I* showed the most significant cell proliferation rate. Between 5 and 20 hours, primary skeletal muscle cells proliferated at the greatest rate, this can be seen by the step gradient in the curve in *Proliferation Phase I* in Fig 2A, displaying mean cell index over time (hours) for each breed. Following this initial acceleration of growth between 5 and 20 hours, proliferation slowed (gradient is not as steep). This was the second phase of growth, termed *Proliferation Phase II* and occurred from 20 hours onwards, refer Fig 2B. Rates of cell proliferation for individual animals through the two growth phases were calculated as the slope of the growth curve. Differences in cell proliferation between breeds during *Proliferation Phase I* and *Proliferation Phase II* were different, as shown in Fig 3A and Fig 3B respectively. The rate of cell proliferation for Angus (0.111) was found to be higher than cells from Hereford (0.065) and WagyuX (0.047) during *Proliferation Phase I* (*P* < 0.05). This also occurred during *Proliferation Phase II* (*P* < 0.05), where cells from Angus (0.033) cattle displayed a higher proliferation rate than cells from Hereford (0.027) and WagyuX (0.022) cattle.

**Fig 4:**
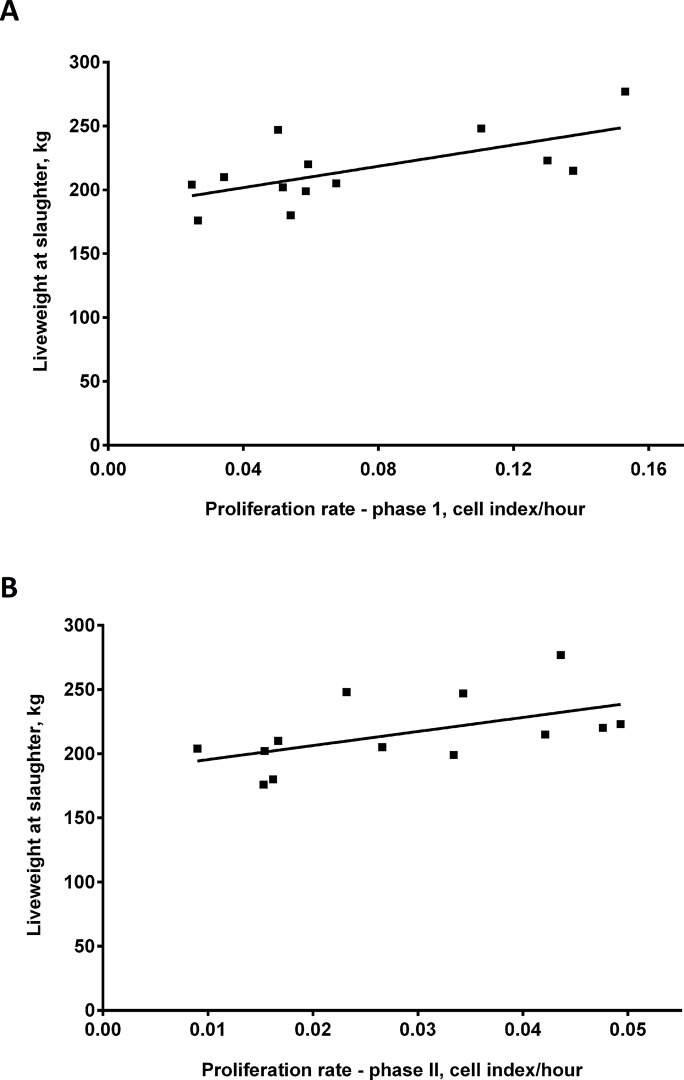
Proliferation rate of *bovine* primary skeletal muscle cells *in vitro* is positively correlated with liveweight of cattle at slaughter. A. Rate of proliferation of *bovine* primary skeletal muscle cells *in vitro* versus liveweight of cattle at slaughter (6-months old) for Phase I (5–20 hours in culture)(R^2^ = 0.43) and B. Phase II (20–72 hours in culture)(R^2^ = 0.30). P < 0.05 for linear regressions in A and B.

**Fig 5:**
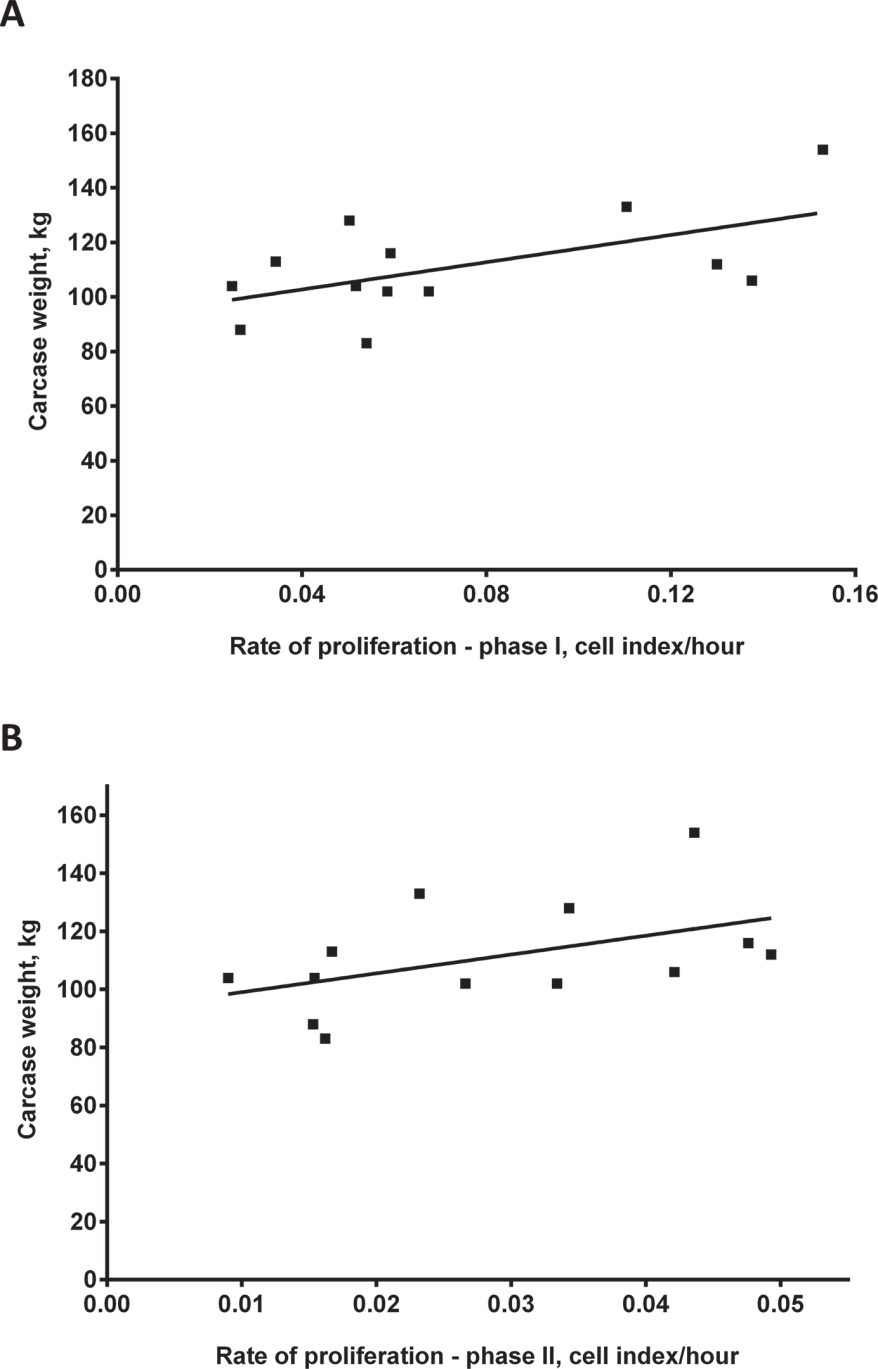
Proliferation rate of *bovine* primary skeletal muscle cells *in vitro* positively correlates with carcase weight. Rate of proliferation of *bovine* primary skeletal muscle cells *in vitro* versus carcase weight (6-months old) for A. Phase I (5–20 hours in culture)(R^2^ = 0.34) and B. Phase II (20–72 hours in culture)(R^2^ = 0.23)(B). P < 0.05 for linear regressions in A and B.

A significant positive linear regression was found between the rate of myoblast proliferation and the liveweight of cattle at slaughter and lean meat yield (carcase weight)(at 6 months of age)(*P <* 0.05). This suggests that the higher the rate of proliferation of myoblasts *in vitro* the greater the liveweight and lean meat yield from cattle at 6 months of age. Refer to Fig 4 for linear regressions with liveweights and Fig 5 for regressions with carcase weights. Individual liveweights and carcase weights of animals are also shown in Table 2.

**Table 2:** Liveweights (kg) and carcass weights (kg) of 6 months old Angus (n = 4), Hereford (n = 5) and Wagyu X Angus (n = 4) Angus cattle sampled for *bovine* primary skeletal muscle cells^1^.

*Breed*	Animal ID, kg	Liveweight at slaughter, kg	Carcass weight, kg
Angus	6–7211	247	128
Angus	7–7212	248	133
Angus	10–7215	223	112
Angus	13–7218	277	154
Hereford	2–7207	199	102
Hereford	3–7208	210	113
Hereford	5–7210	176	88
Hereford	8–7213	205	102
Hereford	15–7220	215	106
Wagyu X Angus	4–7209	180	83
Wagyu X Angus	9–7214	220	116
Wagyu X Angus	11–7218	202	104
Wagyu X Angus	12–7217	204	104

^1^Data was kindly obtained Casino abattoir near Lismore, New South Wales. Australia.

### Gene Expression of Myogenic Gene Markers in *bovine* Primary Skeletal Muscle Cells

**Fig 6:**
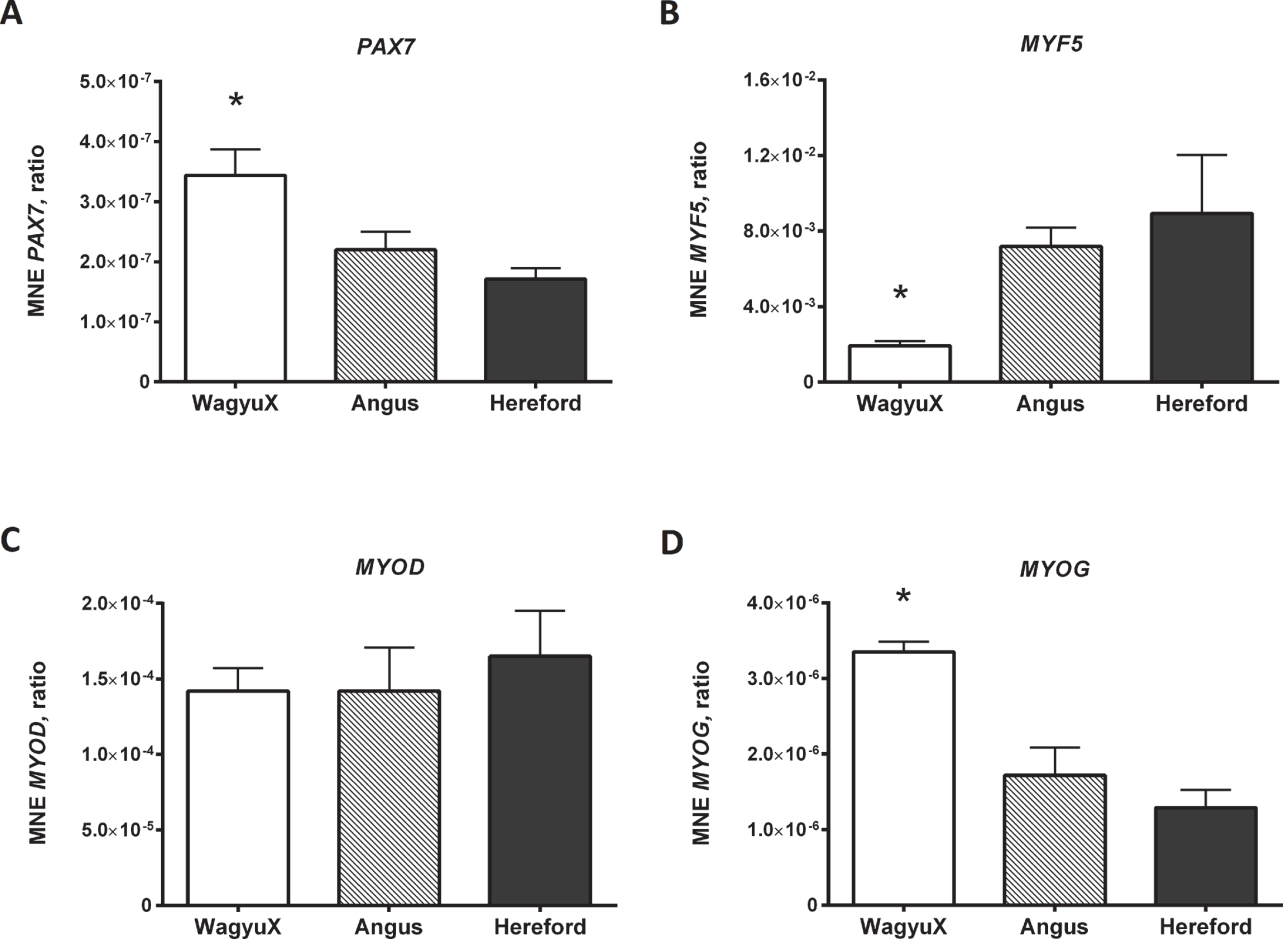
Expression of muscle regulatory factors in un-differentiated *bovine* primary skeletal muscle cells isolated from Angus, Hereford and WagyuX cattle. Expression of *PAX7* (A), *MYF5* (B), *MYOD* (C) and *MYOG* (D) are expressed as mean normalised gene expression (**MNE**) using *bovine HPRT* (hypoxanthineguanine phosphoribosyltransferase) as the housekeeping gene. Error bars represent SEM.

Gene expression of myogenic markers, *MYOD*, *MYF5*, *MYOG* and *PAX7*, were determined in myoblasts (90% confluent) (Fig 6). Gene expression of *MYF5* in primary muscle cells was found to be lower in WagyuX than that of Angus and Hereford cells (*P <* 0.05). However, gene expression of *PAX7* and *MYOG* was found to be higher in primary muscle cells from WagyuX cattle compared to cells from Angus and Hereford (*P <* 0.05). No significant difference was found in the gene expression of *MYOD* in myoblasts between WagyuX, Angus and Hereford cattle, suggesting no difference in the myogenic nature of cells isolated from these breeds.

## Discussion

Primary skeletal muscle cells isolated from 6 month old Angus, Hereford and WagyuX cattle showed different rates of proliferation *in vitro*. The mean rate of proliferation of myogenic cells was higher in Angus, compared with primary muscle cells from Hereford and WagyuX animals. Regression analysis found the rate of proliferation (slope analysis) to be positively related to liveweight and carcase weights. This experiment highlights that increased liveweights and carcase weights found in Angus cattle are influenced by mechanisms controlling proliferation of activated satellite cells and myoblasts. A similar study by Lavulo et al. [7] found myoblasts that were isolated from the *Semitendinosus* (ST) muscle of Callipyge sheep, which display a hypertrophy muscle phenotype, proliferate faster than myoblasts isolated from normal ST muscle. These authors suggest the increased proliferative growth of myoblasts isolated from Callipyge sheep may be playing a significant role in hypertrophy of muscles displaying this phenotype. This further suggests growth of meat producing animals may be influenced largely by the proliferation of myoblasts and other cells *in vivo*, offering an opportunity to manipulate myogenesis on the cellular level to increase muscle mass in cattle and thus produce greater beef yield.

Gene expression of *MYF5* in myoblasts of WagyuX cattle was down-regulated compared with myoblasts from Angus cattle. *MYOD* gene expression was not different between breeds. *MYOD* and *MYF5* are part of the myogenic basic HLH transcription factor family of genes, expressed in undifferentiated proliferating myoblasts and myotubes and are required for myogenic determination of muscle precursors and maintenance of myogenic cell populations [2,9,1]. Reduced proliferation in primary skeletal muscle cells in WagyuX cattle may have occurred due to low *MYF5* levels playing a role in maintenance of the satellite cell pool. Yoshida et al. [12] found the generation of reserve cells (satellite cells) in C2C12 cells, occurred with low expression of *MYF5*, suggesting a role for *MYF5* in maintaining satellite cell populations. Thus it is possible that down-regulation of *MYF5* in *bovine* primary skeletal muscle cells from WagyuX cattle may have contributed to reduced proliferation in myoblasts during *Proliferation Phase I*, as low levels of *MYF5* acted to maintain quiescent satellite cell pools and reduced the number of satellite cells that were activated to proceed as myoblasts. This may also explain why *PAX7*, a marker of satellite cells, expression was higher in cells from WagyuX cattle.

Low *MYF5* levels in WagyuX primary cell cultures may also influence development of intramuscular adipocytes and the high marbling phenotype well known in Wagyu cattle. Tajbakhsh et al. [10] found muscle precursors in *Myf5* null mice remained multipotent and adopted an alternate fate, differentiating into other somatic lineages, including adipocytes, dependent on their local enviroment. Gayraud-Morel et al. [3] found that *Myf5* null mice showed increased accumulation of adipocytes in the regeneration bed following freeze induced injury. Kablar et al. [4] also found increased accumulation of adipose tissue in the absence of myoblasts and differentiated skeletal muscle in newborn *Myf5* null mice. *Myf5* null satellite cell cultures also converted into adipoctyes, but this was at low frequency [3]. Thus, it is possible that low expression of *MYF5* in Wagyu cattle causes myogenic precursors to adopt an alternative fate, such as the adipogenic lineage, which would ultimately increase IMF deposition in these cattle.

*PAX7* was up-regulated in myoblasts from WagyuX cattle compared with Hereford cattle. Up-regulation of *PAX7* in myoblasts from WagyuX cattle could be due to reduced *MYF5* expression, decreasing the number of satellite cells activated into the myogenic lineage and establishment of an alternative pathway which induces adipogenesis as mentioned above. Down-regulation of *MYF5* in Wagyu cattle could cause satellite cells to trans-differentiate into adipocytes (white adipose tissue), increasing their adipogenic potential and thus IMF deposition. Kook et al. [6] found satellite cells isolated from muscle of adult Hanwoo cattle were able to trans-differentiate in adipocytes (induced by rosiglitazone).

*MYOG* expression was also differentially expressed in primary muscle cells from WagyuX cattle, compared with cells from Angus and Hereford. *MYOG* was up-regulated in cells from WagyuX when compared to cells from Angus and Hereford. Thus *MYOG* activation appears to occur much earlier in WagyuX myoblasts. *MYOD* is known to activate *MYOG* to induce differentiation of myoblasts [5]. Down-regulation of *MYF5* in WagyuX myoblasts may cause *MYOG* to be activated at an earlier stage. *MYF5* is known not to be involved in activating *MYOG* but may actually play a role delaying its activation because of its function in satellite cell activation and expansion. Investigations on *Myf5* null and *Myf5*:*Myod* null mice showed delayed myogenic differentiation, which does not support this [3,4]. However, muscle satellite cells grown in culture from these *myf5* null mice did not show any difference in differentiation compared with the control. Thus the theory that *MYF5* could somehow be involved in delaying *MYOG* activation in cattle cannot be ruled out. Further investigations are required to test this.

## Conclusion

This investigation found that primary skeletal muscle cells from Angus, Hereford and WagyuX have different rates of proliferation *in vitro*, providing greater insight into possible explanations as to why Angus, Hereford and WagyuX cattle have phenotypic differences observed in skeletal muscle. Myoblasts from Angus cattle proliferated at a higher rate (approximately twice as fast) compared with myoblasts from WagyuX cattle. The proliferation rate was also higher in cells from Angus cattle compared with myoblasts from Hereford cattle. The proliferation rate of primary skeletal muscle cells from these three breeds of cattle were also found to be positively related to liveweight at slaughter and carcase weight (cattle at 6 months of age). Differential gene expression was also found in cells between breeds in transcription factors vital for myogenesis. Down-regulated *MYF5* and up-regulation (or possibly early activation) of *MYOG* gene expression in WagyuX myoblasts (compared to cells of Angus and Hereford) may be playing a role in influencing the reduced muscling phenotype observed in Wagyu cattle.
